# Low-Cost Approaches in Neuroscience to Teach Machine Learning Using a Cockroach Model

**DOI:** 10.1523/ENEURO.0173-24.2024

**Published:** 2024-12-12

**Authors:** Vincent Truong, Johnathan E. Moore, Ulises M. Ricoy, Jessica L. Verpeut

**Affiliations:** ^1^Department of Psychology, Arizona State University, Tempe, Arizona 85287; ^2^Department of Neuroscience, University of Arizona, Tucson, Arizona 85721

**Keywords:** addiction, behavior, cockroach, kinematics, machine learning, teaching

## Abstract

In an effort to increase access to neuroscience education in underserved communities, we created an educational program that utilizes a simple task to measure place preference of the cockroach (*Gromphadorhina portentosa*) and the open-source free software, SLEAP Estimates Animal Poses (SLEAP) to quantify behavior. Cockroaches (*n* = 18) were trained to explore a linear track for 2 min while exposed to either air, vapor, or vapor with nicotine from a port on one side of the linear track over 14 d. The time the animal took to reach the port was measured, along with distance traveled, time spent in each zone, and velocity. As characterizing behavior is challenging and inaccessible for nonexperts new to behavioral research, we created an educational program using the machine learning algorithm, SLEAP, and cloud-based (i.e., Google Colab) low-cost platforms for data analysis. We found that SLEAP was within a 0.5% margin of error when compared with manually scoring the data. Cockroaches were found to have an increased aversive response to vapor alone compared with those that only received air. Using SLEAP, we demonstrate that the *x*–*y* coordinate data can be further classified into behavior using dimensionality-reducing clustering methods. This suggests that the linear track can be used to examine nicotine preference for the cockroach, and SLEAP can provide a fast, efficient way to analyze animal behavior. Moreover, this educational program is available for free for students to learn a complex machine learning algorithm without expensive hardware to study animal behavior.

## Significance Statement

This method demonstrates a novel utilization of machine learning using free cloud-based programming to analyze cockroach behavior in a linear track. This educational program can be implemented in the classroom as a low-cost tool to teach neuroscience and machine learning. Further, implementing these computational tools allows students to explore important questions in behavioral neuroscience, such as learning and memory, drug seeking, and exploratory locomotor behavior.

## Introduction

A lack of proper educational tools and financial resources prevents students and trainees from grasping the skills required for neuroscience research and learning (Kezar and Elrod, 2012). Disparities in undergraduate neuroscience education are a growing concern (Rochon et al., 2019; Hoy, 2021) in rural areas where postsecondary education in STEM fields presents unique challenges that contribute to lower attainment rates and discourage minorities from participating in science (Strasser et al., 2016; Kricorian et al., 2020; O’Neal and Perkins, 2021). Lack of funding, costly recording equipment, insufficient computational power, and extensive training requirements for protocol clearance are barriers to accessing neuroscience research (Stanford, 2023). In particular, machine learning algorithms that can tackle large-scale data common in neuroscience often require a costly graphic processing unit (GPU). To alleviate these disparities, free cloud-based platforms, such as Google Colab, can process machine learning algorithms without the need for a GPU and have been used for several educational tools, including genomics, thermodynamics, and molecular studies (Arantes et al., 2021; Poolman et al., 2022; Vallejo et al., 2022).

Additional attempts to increase engagement for rural students include using accessible, low-cost experimental models like the cockroach (Torres et al., 2021). Using the cockroach, students can implement a variety of experiments to analyze neurophysiology and behavior without extensive equipment training or institutional research clearance for traditional vertebrate models. However, traditional methods of behavior analysis involve hand-scoring tracking data from video recordings, which consumes a significant amount of time, requires behavior experts, and introduces room for inconsistency in behavior categorization. Thus, we implemented a free, open-source machine learning pose tracking software, Social LEAP Estimates Animal Poses (SLEAP; Pereira et al., 2019, 2022), to analyze kinematic data from video recordings of cockroaches in a linear maze. SLEAP allows for automated tracking of animal movement and poses for an unlimited number of videos (Klibaite et al., 2022; Verpeut et al., 2023). SLEAP does not require any previous coding experience and can easily be utilized through an intuitive graphical user interface, increasing receptivity and confidence for individuals new to neuroscience.

As an introduction to the rigorous research process involved in neuroscience, our modular education program allows students to create and execute original experiments with cockroaches, measure kinematics using SLEAP, and analyze data using Google Colab. This beginner-friendly program will empower students with sophisticated tools that will prepare them to tackle large-data research in future endeavors. The aims of this program include the following: (1) empower students of rural backgrounds with foundational research skills; (2) introduce computational competency for students in preparation for a career in neuroscience or related fields; and (3) address economic disparities by providing low-cost tools. To test this novel method, cockroach behavior was examined in a linear track with exposure to air, vapor, or vapor plus nicotine. Manual or hand-scored data was compared with SLEAP to evaluate behavior and software was created on Google Colab for data analysis. We demonstrated this program during two workshops and found a self-reported 94% increase in behavior tracking knowledge using SLEAP and over half have incorporated SLEAP into their research. These results suggest that this program allows for detailed analysis of cockroach behavior in a simple linear track, and our newly designed educational program can be used to teach machine learning using simple cost-effective strategies.

## Materials and Methods

### Educational design

This program is designed to allow K-12 and college undergraduate students access to neuroscience and biological education, as well as a hands-on experience with examining behavior in an invertebrate model using machine learning. Students are often not exposed to these experiences in neuroscience until higher levels such as graduate and PhD programs. Our results demonstrate that SLEAP can go beyond manual analysis and have designed a program to enhance and improve core scientific standards in Arizona and beyond (Table 1). The program structure includes example datasets, videos, and a lecture on performing model training and tracking of cockroach nodes as the animal walks down a linear track. Schematics are included to replicate the linear track (Extended Data Fig. 1-1) and syringe pump to deliver air, vape, or nicotine aerosolized solutions. The machine learning program, SLEAP, can be downloaded to any computer, and analysis can be performed using Google Colab. This strategy of using open-source free code increases equality access for all students.[Table T2]

**Table 1.** Arizona educational standards

Arizona essential knowledge and skills	Essential question	Activities
Arizona Science Standards Core Ideas for Using Science: U1	U1: Scientists explain phenomena using evidence obtained from observation and or scientific investigations. Evidence may lead to developing models and or theories to make sense of phenomena. As new evidence is discovered, models, and theories can be revised.	Observing cockroach behavior in different experimental settings to evaluate the effects of said environmental changes on behavior.
Arizona Science Standards Science and Engineering Practices	The science and engineering practice describe a robust process for how scientists investigate and build models and theories of the natural world or how engineers design and build systems.	Develop new questions and design novel experimental apparatus to investigate questions surrounding observed behaviors.
Arizona Science Standards Core Idea of Life Science: L2	Organisms require a supply of energy and materials for which they often depend on, or compete with, other organisms.	Observe and design experiments to explore ideas of how different substances impact behavior (i.e., nicotine).

**Table 2. T2:** Experimental conditions

	Males	Females
Negative control: air	3	3
Positive control: vape only	3	3
Experimental: vape plus nicotine	3	3

The structure of this program begins with an introduction to using SLEAP with a step-by-step tutorial. Example videos, complete datasets, trained models, and analysis pipelines are included for students to follow. Activities include labeling the cockroach nodes in the video, training the model, running inference to label each node across all frames, creating figures to represent the data, and acquiring quantitative results. These quantitative results include distance traveled, time spent in each quadrant, statistical analysis between treatment groups, velocity, and clustering for behavioral phenotypes. After completion of this program, students will have learned the basics of testing invertebrate behavior, designing an experiment, machine learning, figure creation, statistical analysis, and Python. It is recommended that students are assessed for their knowledge before and after the program to evaluate learning and impact.

### Experimental design

**Figure 1.**
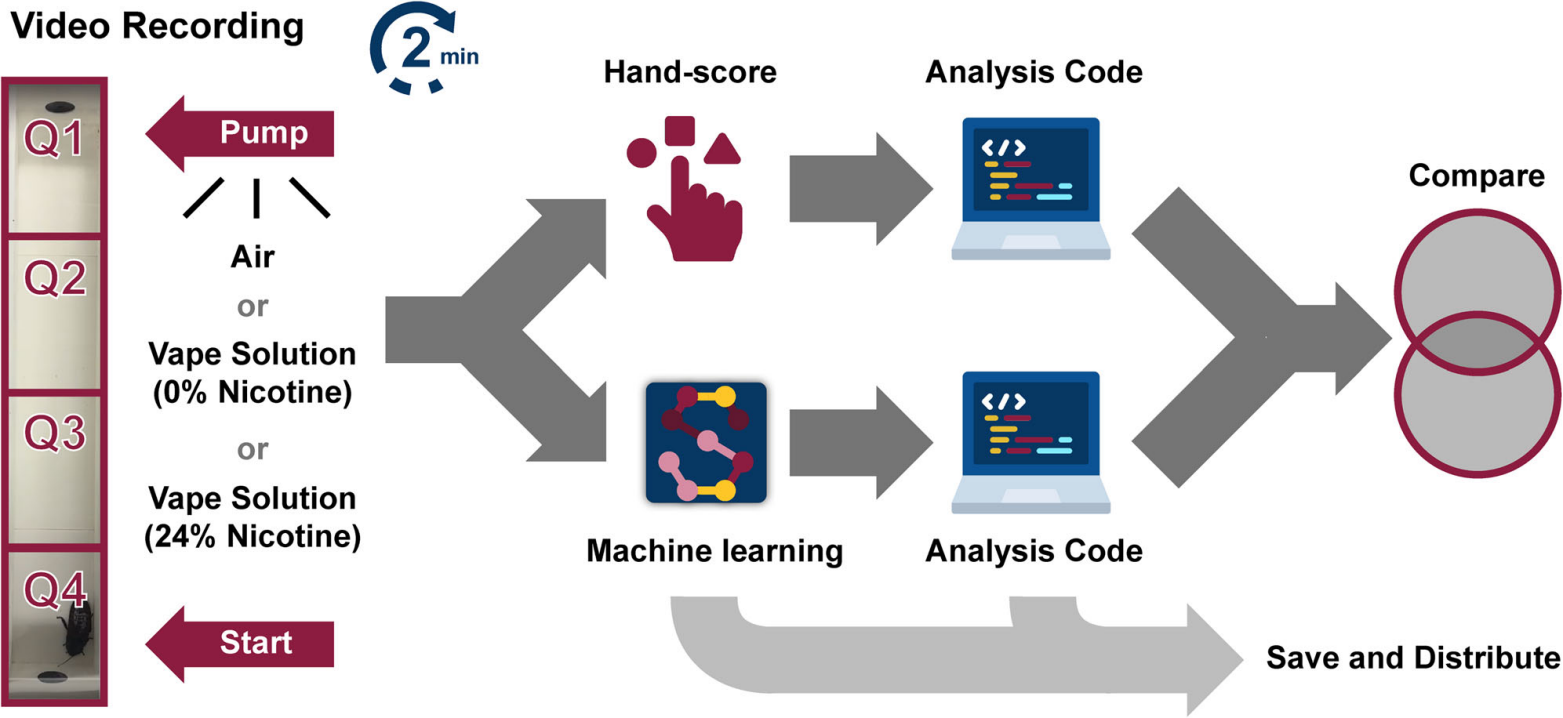
Analysis pipeline for cockroach behavior. Cockroaches were video recorded in a linear track and exposed to air, a 0% nicotine vape solution, or a 24% nicotine vape solution for 2 min. Time in different quadrants, movement, velocity, and behavior were scored either by hand and/or through machine learning techniques (SLEAP), analyzed and compared in Google Colab. SLEAP was within a 0.5% margin of error when compared with hand-scored data and could find unique aspects of behavior that were not possible to analyze by eye (i.e., speed and distance traveled of the cockroach). See Extended Data Figure 1-1 for more details.

*Gromphadorhina portentosa* cockroaches were used to replicate a classic addiction paradigm (Crespi, 1942; Ettenberg, 2009; Espinoza et al., 2022; Hull, n.d.). Each experimental group (*n* = 6) was housed in separate living environments at room temperature on a normal 12 h light/dark cycle (lights on between 8:00 A.M. and 8:00 P.M.). All groups had access to nourishment *ab libitum*. The UTEP Institutional Animal Care and Use Committee approved all procedures. Three groups of six roaches each (*n* = 18 between 8 and 12 months old; Table 2) were tested for free locomotion in a 4×24 in 3-D printed runway model chamber. Roaches that did not survive were removed from the paradigm, specifically one female in the control group and one male/one female in the 0% nicotine group. A negative control group received a pump of room air to compare against confounds (i.e., pressure and sound), whereas a positive control group received a vape solution containing Kosher USP grade propylene glycol and kosher USP grade vegetable glycerin in at 50:50 ratio PG/VG. The experimental group received a vape solution containing 99.9% pharmaceutical grade freebase 24% nicotine in 50:50 PG/VG sourced from Vapor Vapes. All subjects were placed in the first quadrant (Fig. 1) and allowed to freely move in the chamber for 2 min while recorded with an iPhone X camera, recording 1080p HD video at 60fps from a top-down perspective. Video files were stored for hand-scoring and machine learning training (SLEAP) for time in different quadrants, movement, velocity, and behavior. Each group was tested between 9 A.M. and 9 P.M. for 14 d.

10.1523/ENEURO.0173-24.2024.f1-1 Figure 1-1 Customized apparatus for testing cockroach behavior after exposure to air, vape, or nicotine. **A)** Linear track measuring 24 in x 4 in x 2.5 in (length x width x height). **B)** Syringe pump connected to apparatus that delivers vape to the linear track. Download Figure 1-1, TIF file.

### SLEAP model evaluation

After labeling and training on 348 video frames, validation of the SLEAP model indicated a 6.8% false-positive rate, 2.0% false-negative rate, 84.0% true-positive rate, and 7.2% true-negative rate. This model had 97.7% recall ability and 92.5% precision ability. All 227 video files were able to be processed by SLEAP to generate time-dependent pose data and were all compatible with our analysis code (Movie 1). Locomotion-heavy nodes (i.e., antennas) or easily obscured nodes (i.e., hind leg joints) yielded higher error rates during pose estimation. However, stable anatomy reference points, such as the head, had an average pose estimation error of <5 pixels from the ground truth.

### Hand-scoring and machine learning scoring

**Figure 2.**
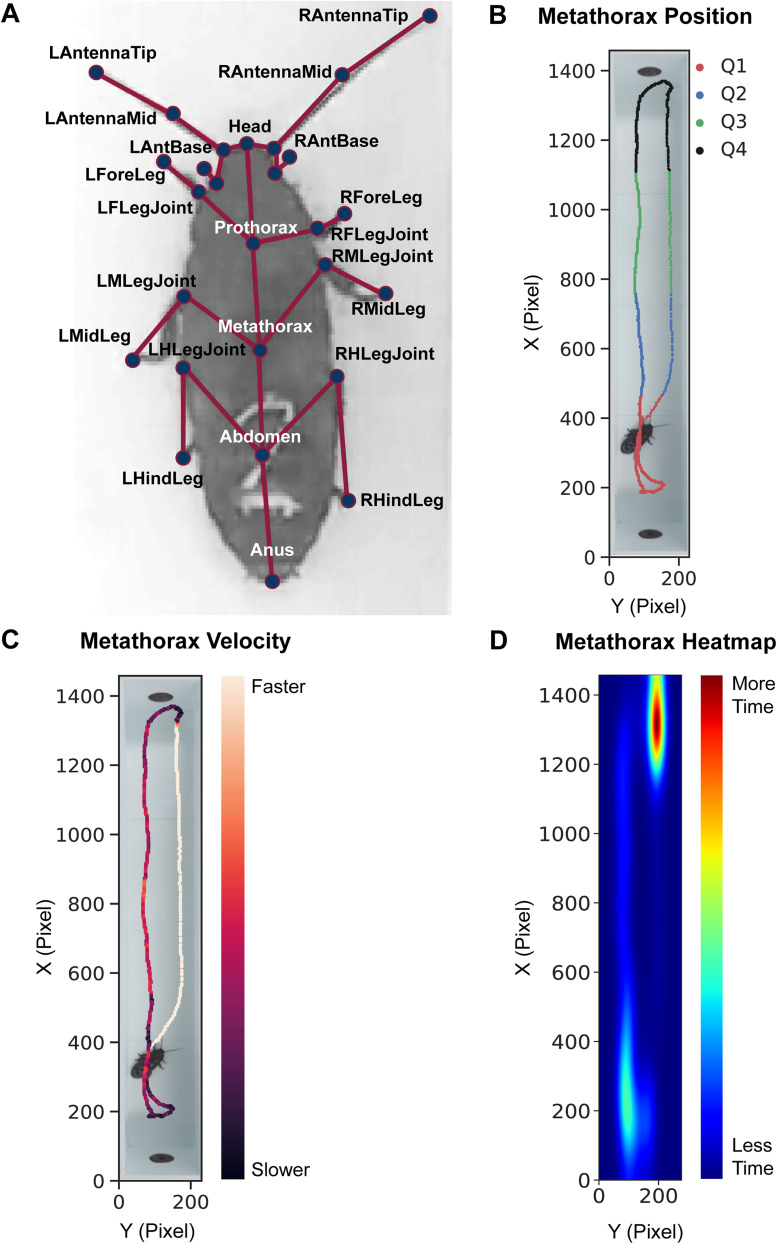
SLEAP to track cockroach behavior. ***A***, A sample skeleton overlay on top of a single video frame. “R” and “L” denote the “Right” and “Left” sides, respectively. Discrete anatomical points were chosen to showcase the complexity that SLEAP is capable of handling. ***B***, Metathorax tracks in each quadrant of the linear track could be separated using analysis code of *x* and *y* coordinates from SLEAP. This method could quantify the amount of time and distance traveled per quadrant. ***C***, Metathorax tracks shown with velocity. The speed of the roach is slower by the port than the rest of the linear track. ***D***, A heat map representation of metathorax tracks across a single video. Warmer colors indicate more time spent in a certain area. Cooler colors represent less time spent in a certain area.

For each session recording, cockroaches were scored in 1 s intervals by a single scorer and each second was given a score from 1 to 4, indicating the position of the roach's head in the corresponding quadrant. Scoring was repeated using a machine learning roach model created with Social LEAP Estimates Animal Poses (SLEAP). Discrete, well-defined anatomy joints (Gupta et al., 1990) were chosen as nodes to reduce ambiguity and promote clear placement criteria during pose fitting of the skeleton. Nodes of interest (27 total) included the head, antenna base, antenna midpoint, antenna tip, palp base, palp tip, prothorax, metathorax, abdomen, anus, fore leg (joint and tip), mid leg (joint and tip), and hind leg (joint and tip; Fig. 2*A*). Bilateral anatomical parts had separate nodes for the right and left sides. We selected 27 nodes to capture sensitive behaviors specific to the cockroach model; however, the number of nodes should be selected to answer specific experimental questions. For instance, to capture the curvature of antenna movements to assess potential grooming behavior, three nodes were required. However, these may be omitted if grooming was not of interest. Joints obscured by the body or blurred due to fast locomotion were omitted from node placement during training. Our model was used to track the position of a single subject across an entire video for all session recordings (Fig. 2*B*). Python code was then used to disseminate velocity (Fig. 2*C*) and a heat map of time spent in locations across the linear track (Fig. 2*D*) from SLEAP. To mimic the criteria for hand-scoring, the head node was used as the anchor point in our analysis code when determining the length of time the roach spent in certain quadrants. This pipeline was repeated for all roaches.

### Creation of analysis pipeline

The SLEAP Starter Kit contains nine files that include the following: (1) an instruction manual (“0_START_HERE”), (2) a presentation to use in the classroom (“1_SLEAP_Presentation”), (3) a sample video (“2_SampleVideo.mp4”), (4) SLEAP skeleton for the cockroach (“3_Cockroach_skeleton.json”), (5) labeled SLEAP file (“4_SampleSLEAPFile.slp”), (6) trained SLEAP models of centroid and centered (“5_Trained_models”), (7) compressed final SLEAP data file (“6_Sample_h5_file.h5”), (8) Colab analysis (“7_Roach_Analysis_Colab”), and (9) SLEAP final excel datasheet (“8_Example_Datasheet”). Users can access these files and walk through the instruction manual to analyze either the sample data provided or user-obtained data.

In Google Colab, the analysis pipeline is organized in code blocks for intuitive navigation. Users can run the code by pressing the play icon for each section to execute the code block to run the analysis. The only changes required are the file locations, which must be changed if novel data is used to match the file location of the user. In the Google Colab, there are five main sections. Section 1 downloads the necessary Python packages for analysis. Section 2 is an optional block that can generate inference files using the pre-existing model. Section 3 is used to load the inference files and restructured data obtained from SLEAP outputs for analysis in Section 4. Lastly, Section 5 recreates all plots found in this manuscript. The code is annotated and comments are included to explain the purpose of each section, appropriate parameters to adjust, and necessary variables to change. All code used for visualizations is available through Github or Linktree (https://linktr.ee/sleapingroaches) for educators and students to utilize, along with a starter guide that explains how to utilize example files.

### Statistical analysis

All statistics were performed using Python 3 (statsmodels, pingouin, scipy, matplotlib, numpy) in Google Colab. Data are presented as mean ± SD unless otherwise stated. To decide parametric or nonparametric analysis of group means, normality and homogeneity of variances were tested with the Shapiro–Wilk and Levene's test, respectively, before proceeding with further statistical analysis. If either the Shapiro–Wilk test or Levene's test was significant, the nonparametric Kruskal–Wallis test was performed to compare metrics (i.e., time or distance) between quadrants or groups, followed by pairwise comparisons with the Mann–Whitney *U* test (with Benjamini–Hochberg multiple-comparisons correction). Due to a non-normal distribution of data (*p* < 0.001, Shapiro–Wilk) of time spent by roaches in each quadrant, we conducted the nonparametric Kruskal–Wallis and Mann–Whitney *U* test to compare the time spent in a singular quadrant between each group. If parametric tests were warranted, quadrant or group means were compared through mixed-measures ANOVA or one-way ANOVAs with Tukey HSD or Dunnett multiple-comparisons post hoc tests. To compare differences between hand-scored data and SLEAP-scored data, we conducted a paired sample *t* test. Joint positions in *x* and *y* coordinate space were visualized using principal component analysis (PCA) and clustered using *K*-means and/or Uniform Manifold Approximation and Projection (UMAP) with *n* = 2 components and *n* = 42 random states. UMAP is a dimensionality reduction technique to visualize high-dimensional data into distinguishable clusters (Ghojogh et al., 2021). In this case, we used a parametric UMAP, which utilizes neural networks to embed data into a low-dimensional space. The following hyperparameters were used for UMAP: 15 neighbors, 2 components, Euclidean distance, 0.1 minimum distance, 1.0 spread, and 1.0 to preserve the local structure.

## Results

### General behavior

**Figure 3.**
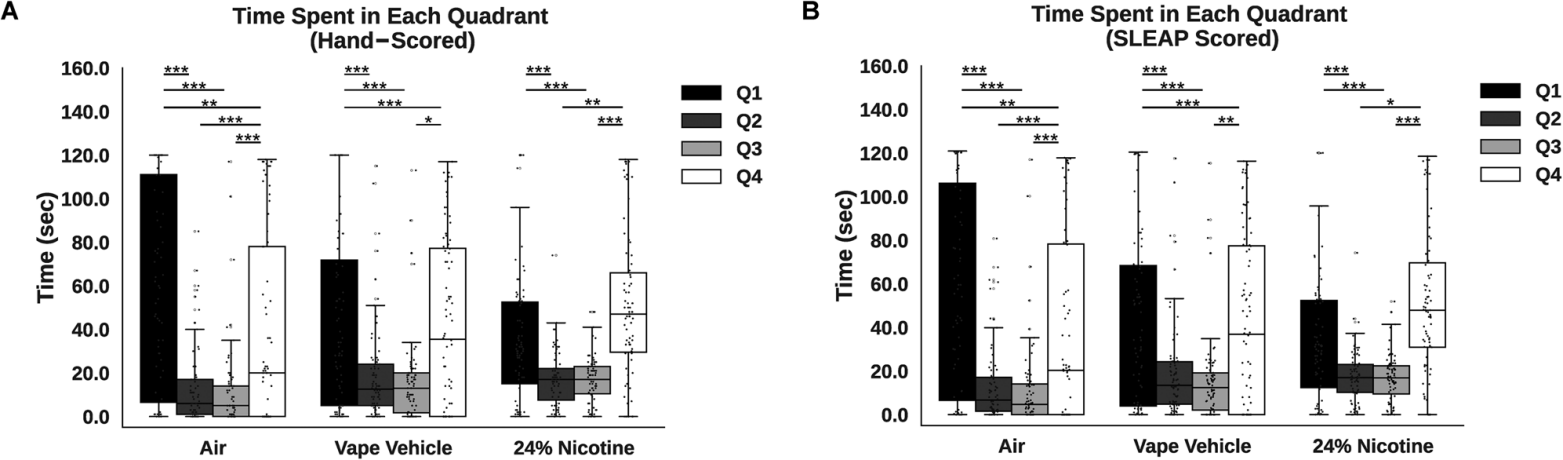
Time spent in each quadrant within groups. ***A***, Recordings were hand-scored to assess time spent in certain quadrants for each roach. For a single roach, scoring was repeated for 14 d and averaged within a quadrant. For roaches receiving air, differences existed in the average amount of time spent between Q1 and Q2 (*p* < 0.001, Mann–Whitney), Q1 and Q3 (*p* < 0.001, Mann–Whitney), Q1 and Q4 (*p* = 0.002, Mann–Whitney), Q2 and Q4 (*p* < 0.001, Mann–Whitney), and Q3 and Q4 (*p* < 0.001, Mann–Whitney). For roaches that received the vape vehicle, significant differences exist in the average amount of time spent between Q1 and Q2 (*p* < 0.001, Mann–Whitney), Q1 and Q3 (*p* < 0.001, Mann–Whitney), Q1 and Q4 (*p* < 0.001, Mann–Whitney), and Q3 and Q4 (*p* = 0.020, Mann–Whitney). For roaches that received 24% nicotine, differences exist in the average amount of time spent between Q1 and Q2 (*p* < 0.001, Mann–Whitney), Q1 and Q3 (*p* < 0.001, Mann–Whitney), Q2 and Q4 (*p* = 0.023, Mann–Whitney), and Q3 and Q4 (*p* = 0.003, Mann–Whitney). ***B***, Recordings were scored with SLEAP to assess time spent in certain quadrants for each roach. For a single roach, scoring was repeated for 14 d and averaged within a quadrant. For roaches that received only air, differences exist in the average amount of time spent between Q1 and Q2 (*p* < 0.001, Mann–Whitney), Q1 and Q3 (*p* < 0.001, Mann–Whitney), Q1 and Q4 (*p* = 0.002, Mann–Whitney), Q2 and Q4 (*p* < 0.001, Mann–Whitney), and Q3 and Q4 (*p* < 0.001, Mann–Whitney). For roaches that received the vape vehicle, differences exist in the average amount of time spent between Q1 and Q2 (*p* < 0.001, Mann–Whitney), Q1 and Q3 (*p* < 0.001, Mann–Whitney), Q1 and Q4 (*p* = 0.001, Mann–Whitney), and Q3 and Q4 (*p* = 0.010, Mann–Whitney). For roaches that received 24% nicotine, differences exist in the average amount of time spent between Q1 and Q2 (*p* < 0.001, Mann–Whitney), Q1 and Q3 (*p* < 0.001, Mann–Whitney), Q2 and Q4 (*p* = 0.025, Mann–Whitney), and Q3 and Q4 (*p* = 0.001, Mann–Whitney).

**Figure 4.**
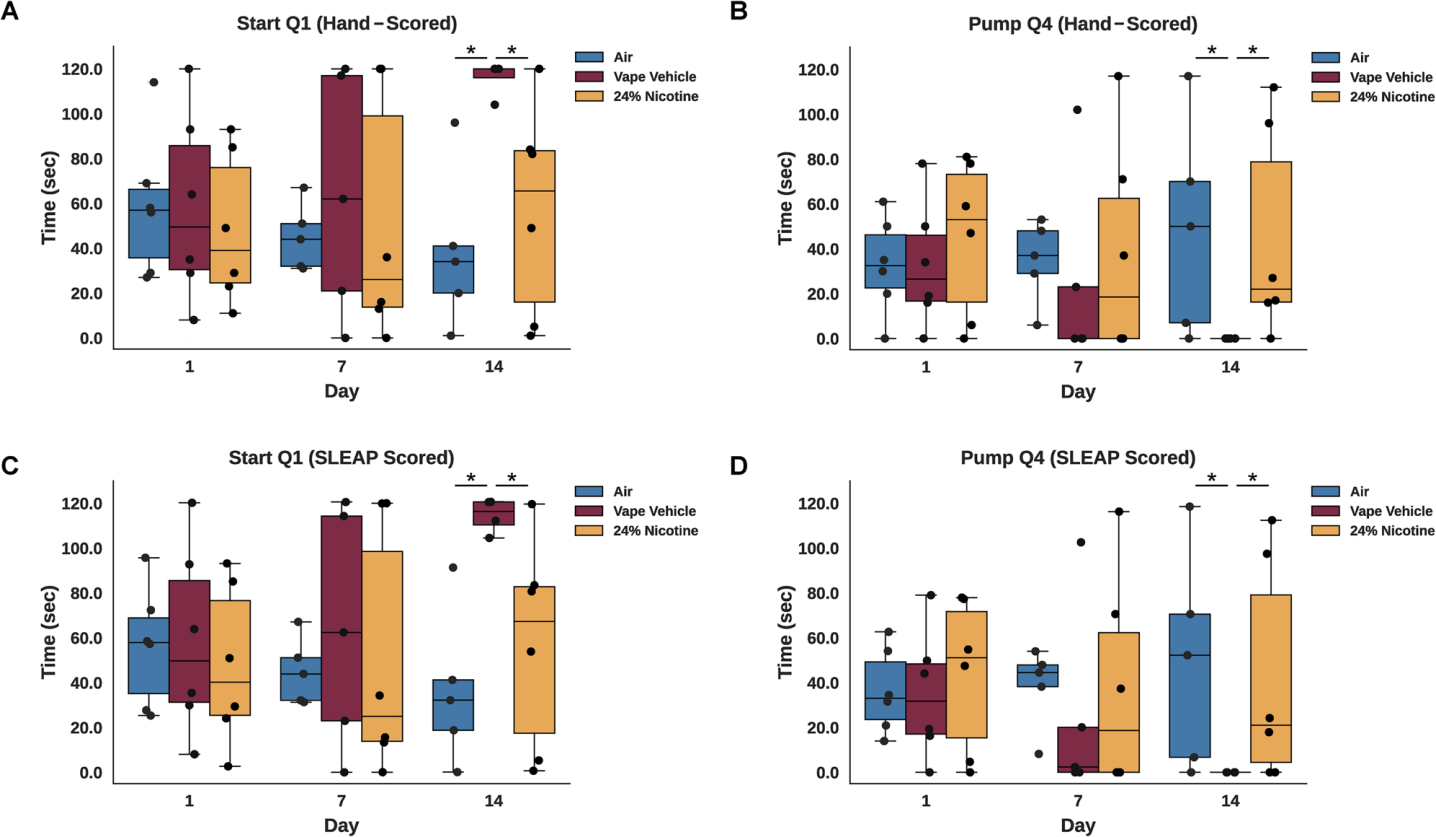
Time spent in each quadrant every 7 d (hand-scored). Video recordings of each roach were hand-scored on the 1st, 7th, and 14th day of the nicotine chamber. Scoring was completed frame by frame in 1 s intervals. ***A***, In quadrant 1 (Q1), no differences were observed in average time spent between groups on Days 1 and 7. On Day 14, roaches exposed to 0% vape solution spent significantly more time in Q1 than roaches receiving air (*p* = 0.018, Mann–Whitney) or 24% nicotine (*p* = 0.048, Mann–Whitney). ***B***, In quadrant 2 (Q2), no differences were observed in average time spent between groups on all days. ***C***, In quadrant 3 (Q3), no differences were observed in average time spent between groups on Days 1 and 14. On Day 7, roaches exposed to 0% vape solution spent significantly less time in Q3 than roaches receiving air (*p* = 0.014, Mann–Whitney). ***D***, In quadrant 4 (Q4), no differences were observed in average time spent between groups on Days 1 and 7. On Day 14, roaches exposed to 0% vape solution spent significantly less time in Q4 than roaches receiving air (*p* = 0.032, Mann–Whitney) or the 24% nicotine group (*p* = 0.023, Mann–Whitney). See Extended Data Figures 4-1 and 4-2 for more details.

**Figure 5.**
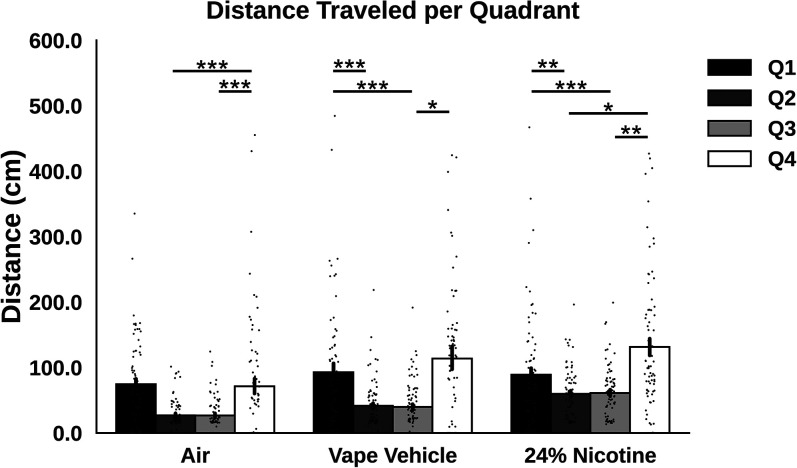
Distance metrics calculated using SLEAP. ***A***, Average distance traveled by each group across 14 d, separated by quartile. Distance was measured using *x*–*y* coordinates obtained from SLEAP and converting pixels into centimeters. For air-exposed roaches, more distance was traveled in Q4 than Q2 (*p* < 0.001, Mann–Whitney). For vape-exposed roaches, distance was traveled in Q1 more than Q2 (*p* < 0.001, Mann–Whitney), Q1 more than Q3 (*p* < 0.001, Mann–Whitney), and Q4 more than Q3 (*p* = 0.020, Mann–Whitney). For nicotine-exposed roaches, distance was traveled in Q1 more than Q2 (*p* = 0.003, Mann–Whitney), Q1 more than Q3 (*p* < 0.001, Mann–Whitney), Q4 more than Q2 (*p* = 0.011, Mann–Whitney), and Q4 more than Q3 (*p* = 0.002, Mann–Whitney) ***B***, Average distance traveled in each quartile across 14 d separated by group. In Q2, air-exposed roaches traveled more than vape-exposed roaches (*p* = 0.001, Mann–Whitney) and nicotine-exposed roaches (*p* < 0.001, Mann–Whitney). Nicotine-exposed roaches also traveled more than vape-exposed roaches (*p* = 0.008, Mann–Whitney). In Q3, air-exposed roaches traveled more than vape-exposed roaches (*p* < 0.001, Mann–Whitney) and nicotine-exposed roaches (*p* < 0.001, Mann–Whitney). Nicotine-exposed roaches also traveled more than vape-exposed roaches (*p* = 0.020, Mann–Whitney). In Q4, only air-exposed roaches traveled more than vape-exposed roaches (*p* < 0.001, Mann–Whitney). ***C***, Stacked bar plots visualizing distance traveled per quadrant between air, vape vehicle, and 24% nicotine.

**Figure 6.**
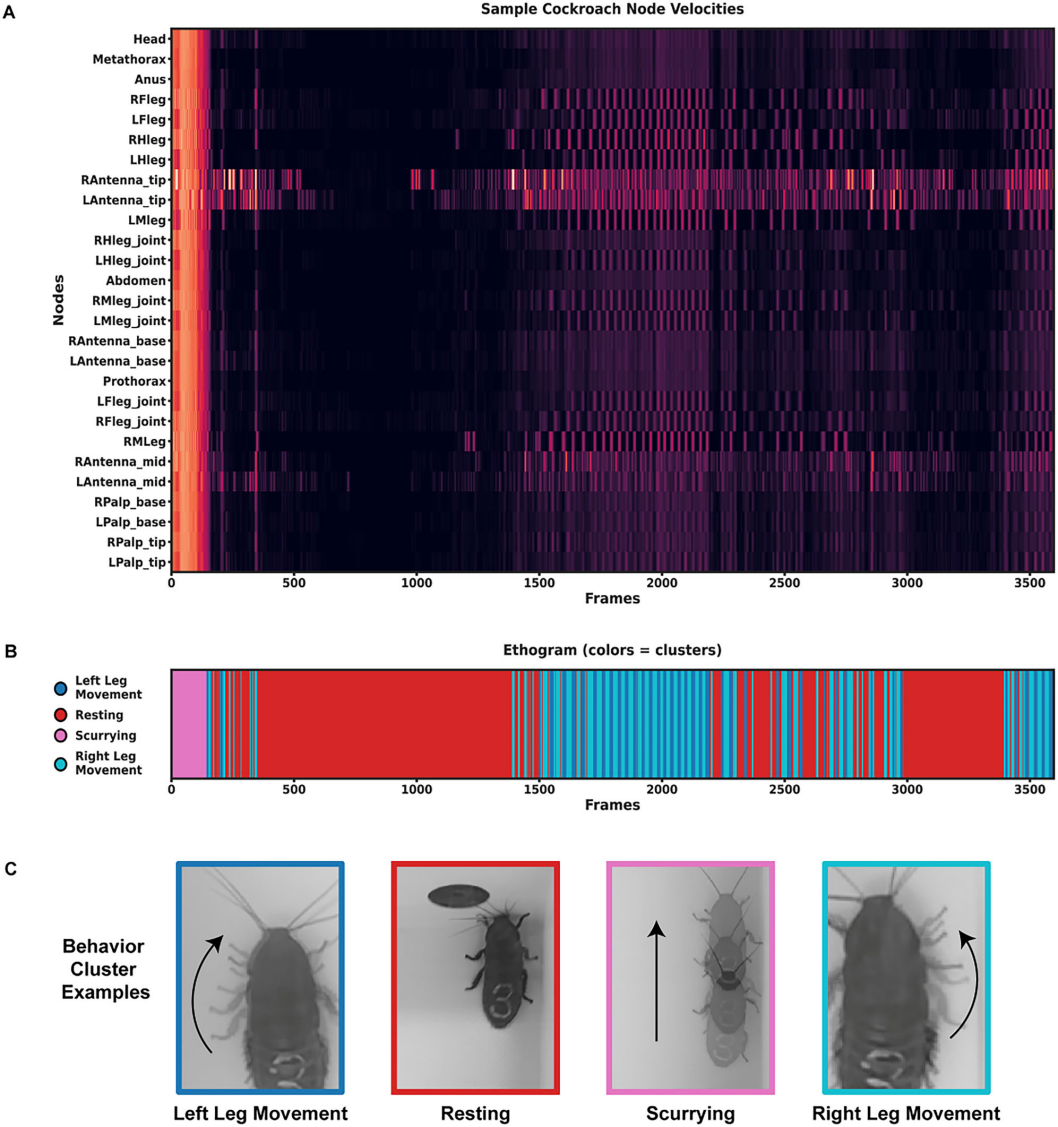
Sample behavior clustering. ***A***, Velocity of all nodes of a singular roach across a 3,600 frame video obtained from SLEAP. Brighter colors indicate faster movement and duller colors indicate slower movement of a particular node in the recording. ***B***, Unsupervised clustering of behavior (*K*-means) using velocity and node position as a clustering basis. Different colors represent different potential behaviors exhibited by the roach (dark blue, left leg movement; red, resting; pink, scurrying; cyan, right leg movement). ***C***, Example frames of each behavior cluster: dark blue for left leg movement, red for resting, pink for scurrying, and light blue for right leg movement. See Extended Data Figure 6-1 for more details.

Overall, in the linear track, roaches spent more time near the start of the chamber in quartile 1 (51.2 ± 2.5 s) or the chamber pump in quartile 4 (34.5 ± 2.4 s; Fig. 3; Fig. 4, Extended Data Fig. 4-1, Extended Data Fig. 4-2). Roaches receiving air would scurry to the pump and either remain there or circle back to the beginning, as shown by our sample roach (Fig. 2*B*). We recommend educators who replicate this experiment to closely evaluate these chambers for potential behaviors of interest. Users can also track non hand-scorable behaviors, such as distance traveled, using our pipeline. Our pipeline detected that roaches receiving air traveled an average of 75.8 ± 8.3 cm in Q1 and 89.9 ± 10.2 cm in Q4 (Fig. 5). Further analysis utilizing the velocity advantage of SLEAP reveals that the metathorax (central body anchor point) traveled at a speed of 2.01 ± 0.19 cm/s for roaches receiving air (Fig. 6*A*, Extended Data Fig. 6-1).

10.1523/ENEURO.0173-24.2024.f4-1 Figure 4-1 Simple linear regression of day versus time spent in a quadrant across 14 days using hand-scored data. We found a simple linear model does not fit our data. **A)** In quadrant 1, roaches exposed to air, vape vehicle, or nicotine display no differences (p = 0.98, ANOVA). Air-exposed roaches resulted in a regression line with the equation y = -0.32x + 37.09 (r^2^ < 0.001). Roaches exposed to vape vehicle resulted in a regression line with the equation y = -0.27x + 60.25 (r^2^ < 0.001). Roaches exposed to 24% nicotine resulted in a regression line with the equation y = 0.50x + 40.41 (r^2^ < 0.001). **B)** In quadrant 2, roaches exposed to air, vape vehicle, or nicotine display no differences (p = 0.93, ANOVA). Air-exposed roaches resulted in a regression line with the equation y = 0.30x + 15.19 (r^2^ < 0.001. Roaches exposed to vape vehicle resulted in a regression line with the equation y = -0.12x + 14.01 (r^2^ < 0.001). Roaches exposed to 24% nicotine resulted in a regression line with the equation y = -0.10x + 19.50 (r^2^ < 0.001). **C)** In quadrant 3, roaches exposed to air, vape vehicle, or nicotine display no differences (p = 0.99, ANOVA). Air-exposed roaches resulted in a regression line with the equation 0.04x + 16.93 (r^2^ < 0.001). Roaches exposed to vape vehicle resulted in a regression line with the equation y = 0.26x + 9.84 (r^2^ < 0.001). Roaches exposed to 24% nicotine resulted in a regression line with the equation y = -0.29x + 17.81 (r^2^ < 0.001). **D)** In quadrant 4, roaches exposed to air, vape vehicle, or nicotine display no differences (p = 0.96, ANOVA). Air-exposed roaches resulted in a regression line with the equation y = -0.04x + 50.83 (r^2^ < 0.001). Roaches exposed to vape vehicle resulted in a regression line with the equation y = 0.13x + 35.89 (r^2^ < 0.001). Roaches exposed to 24% nicotine resulted in a regression line with the equation y = 0.001x + 41.68 (r^2^ < 0.001). Download Figure 4-1, TIF file.

10.1523/ENEURO.0173-24.2024.f4-2 Figure 4-2 Simple linear regression of day versus time spent in a quadrant across 14 days using SLEAP scored data. We found a simple linear model does not fit our data. **A)** In quadrant 1, roaches exposed to air, vape vehicle, or nicotine display no differences (p = 0.92, ANOVA). Air-exposed roaches resulted in a regression line with the equation y = -0.32x + 36.56 (r^2^ < 0.001). Roaches exposed to vape vehicle resulted in a regression line with the equation y = -0.38x + 60.41 (r^2^ < 0.001). Roaches exposed to 24% nicotine resulted in a regression line with the equation y = 0.47x + 39.73 (r^2^ < 0.001). **B)** In quadrant 2, roaches exposed to air, vape vehicle, or nicotine display no differences (p = 0.99, ANOVA). Air-exposed roaches resulted in a regression line with the equation y = 0.27x + 15.56 (r^2^ < 0.001. Roaches exposed to vape vehicle resulted in a regression line with the equation y = -0.14x + 14.45 (r^2^ < 0.001). Roaches exposed to 24% nicotine resulted in a regression line with the equation y = -0.12x + 20.50 (r^2^ < 0.001). **C)** In quadrant 3, roaches exposed to air, vape vehicle, or nicotine display no differences (p = 0.87, ANOVA). Air-exposed roaches resulted in a regression line with the equation 0.13x + 15.70 (r^2^ < 0.001). Roaches exposed to vape vehicle resulted in a regression line with the equation y = 0.35x + 9.58 (r^2^ < 0.001). Roaches exposed to 24% nicotine resulted in a regression line with the equation y = -0.30x + 17.70 (r^2^ < 0.001). **D)** In quadrant 4, roaches exposed to air, vape vehicle, or nicotine display no differences (p = 0.99, ANOVA). Air-exposed roaches resulted in a regression line with the equation y = -0.06x + 52.31 (r^2^ < 0.001). Roaches exposed to vape vehicle resulted in a regression line with the equation y = 0.16x + 36.35 (r^2^ < 0.001). Roaches exposed to 24% nicotine resulted in a regression line with the equation y = -0.06x + 42.39 (r^2^ < 0.001). Download Figure 4-2, TIF file.

### Roaches spend less time in quadrant with vape solution

Cockroach behavior in the linear track was quantified during exposure to air, vape vehicle, or 24% nicotine. In the starting quadrant, Q1, vape-exposed roaches spent more time compared with air-exposed across 14 d, but less time in Q2 (*p* < 0.001), Q3 (*p* < 0.001), and Q4 (*p* = 0.002). Roaches receiving 24% nicotine spent more time in Q2 (*p* = 0.024) and Q3 (*p* = 0.015) compared with vape-exposed, but less time in Q3 compared with the air condition (*p* = 0.022; Fig. 3*A*). Next, using SLEAP, we found similar results, including that vape-exposed roaches spent more time in Q1 (*p* = 0.009) and less time in Q2 (*p* < 0.001) and Q3 (*p* < 0.001) compared with air-exposed roaches (Fig. 3*B*).

Within-group differences of time spent in each quadrant were apparent. Air-exposed roaches spent more time in Q1 and compared with Q2 (*p* < 0.001), Q3 (*p* < 0.001), and Q4 (*p* = 0.002; Fig. 3*A*). The vape vehicle condition spent significantly more time in Q1 compared with Q2 (*p* < 0.001), Q3 (*p* < 0.001), and Q4 (*p* < 0.001). Q3 and Q4 time spent in travel was also significant (*p* = 0.020; Fig. 3*A*). For roaches that received 24% nicotine, there was an opposite effect. Roaches spent more time in Q4 compared with Q2 (*p* = 0.023) and Q4 (*p* = 0.003). Still, more time was spent in Q1 compared with the transition chambers Q2 (*p* < 0.001) and Q3 (Fig. 3*A*). Analyzing this same data with SLEAP, we found similar results. In short, the vape vehicle condition spent significantly more time in Q1 compared with Q2 (*p* < 0.001), Q3 (*p* < 0.001), and Q4 (*p* = 0.001). Nicotine-exposed roaches spent more time in Q4 compared with Q2 (*p* = 0.025) and Q3 (*p* = 0.001; Fig. 3*B*). Overall, roaches exposed to the vape vehicle spent less time in Q4 (Fig. 4*B*), which had the exposure port, while roaches exposed to 24% nicotine spent less time in Q1 (Fig. 4*A*) and more time exploring Q2–4 (Fig. 3).

### Utilizing SLEAP to understand behavior

Using SLEAP, we compared distance traveled, average velocity of nodes, and behavior clustering between groups in each quadrant. Vape-exposed roaches traveled more in Q1 than in Q2 (*p* < 0.001) and Q3 (*p* < 0.001). Roaches who received 24% nicotine spent more time in Q1 than in Q2 (*p* = 0.003) and Q3 (*p* < 0.001). This group also spent more time in Q4 than in Q2 (*p* = 0.011) and Q3 (*p* = 0.002). Cumulatively, air-exposed roaches traveled more than roaches exposed to vape vehicle or 24% nicotine (Fig. 3).

**Figure 7.**
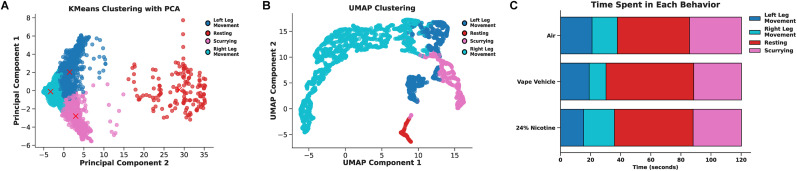
Cluster visualizations. ***A***, Dimensional reduction of *K*-means using principal component analysis (PCA) to capture global structures in clustering behavior. Spatial distance between clusters indicates the degree of difference between behavior clusters. A red “X” indicates the average space for each behavior cluster. ***B***, Visualized dimensional reduction using Uniform Manifold Approximation and Projection (UMAP) to capture local structures in clustering behavior. Spatial distance between clusters indicates the degree of difference between behavior clusters. ***C***, A stacked bar plot of the average time spent in each behavior (left leg movement, right leg movement, resting, scurrying) separated by groups (air, vape vehicle, 24% nicotine). Within any group, the average time resting was significantly more than scurrying (*p* < 0.001, Mann–Whitney), left leg movement (*p* < 0.001, Mann–Whitney), and right leg movement (*p* < 0.001, Mann–Whitney). Roaches exposed to vape vehicle spent more time resting than roaches receiving nicotine (*p* = 0.004, Mann–Whitney).

In addition, the velocity of each node was measured using SLEAP. Air-exposed roaches moved faster compared with vape (*p* < 0.001) or 24% nicotine (*p* < 0.001) conditions (Extended Data Fig. 6-1). Using velocity metrics of each node (Fig. 6*A*, Extended Data Fig. 6-1), we employed one form of unsupervised clustering, *K*-means (Fig. 6*B*). This popular clustering algorithm can partition data into a predefined amount of clusters and those that are closer together are more similar compared with those that are distant. *K*-means was performed for four clusters of behavior, as we observed four main behaviors, including left leg movement, resting, scurrying, and left leg movement. To determine if our observed behaviors were separated via *K*-means, we cross-referenced the clusters with the original recording (Fig. 6*C*). Then, we conducted PCA (Fig. 7*A*), which can be used to simplify complex datasets and to reduce dimensionality of the data by creating new variables or principal components of each *x*–*y* coordinate. To visualize the proximity of behavior clusters, we further transformed clusters into a lower dimensional space using UMAP (Fig. 7*B*) to demonstrate the relationship of the behaviors and identify the underlying structure of the data to find poses that map to behavior. This technique can capture nonlinear relationships that may be missed using PCA but can also handle large datasets more efficiently. Indeed, we found that our observed behaviors could be identified using simple unsupervised clustering. These clusters represent left leg movement (dark blue), resting (red), scurrying (pink), and left leg movement (light blue). Furthermore, when repeating this analysis for all groups to compare behavioral bouts, we find significant differences between behaviors. For instance, roaches from each group typically rest more than scurrying (*p* < 0.001), which would have been tedious to calculate by traditional hand-scoring methods (Fig. 7*C*). This analysis, freely available in our analysis code, showcases just one example of how early researchers can disseminate complex behaviors using beginner-friendly machine learning programs.

10.1523/ENEURO.0173-24.2024.f6-1 Figure 6-1 **Average node velocity.** Velocity of each node was obtained for each roach and averaged within a single group. Velocity was measured using X-Y positional data associated with certain frames obtained from SLEAP metrics. Frames were converted to seconds using the frame per second rate of the camera. **A)** Average node velocity for roaches exposed to air. **B)** Average node velocity for roaches exposed to 0% vape vehicle. **C)** Average node velocity for roaches exposed to 24% nicotine. **D)** Velocity of each node comparing between groups. Averaging all nodes across all days indicates that air-exposed roaches moved faster compared to vape (p < 0.001) or 24% nicotine (p < 0.001) conditions. Download Figure 6-1, TIF file.

### Comparing hand-scored data versus machine learning

**Figure 8.**
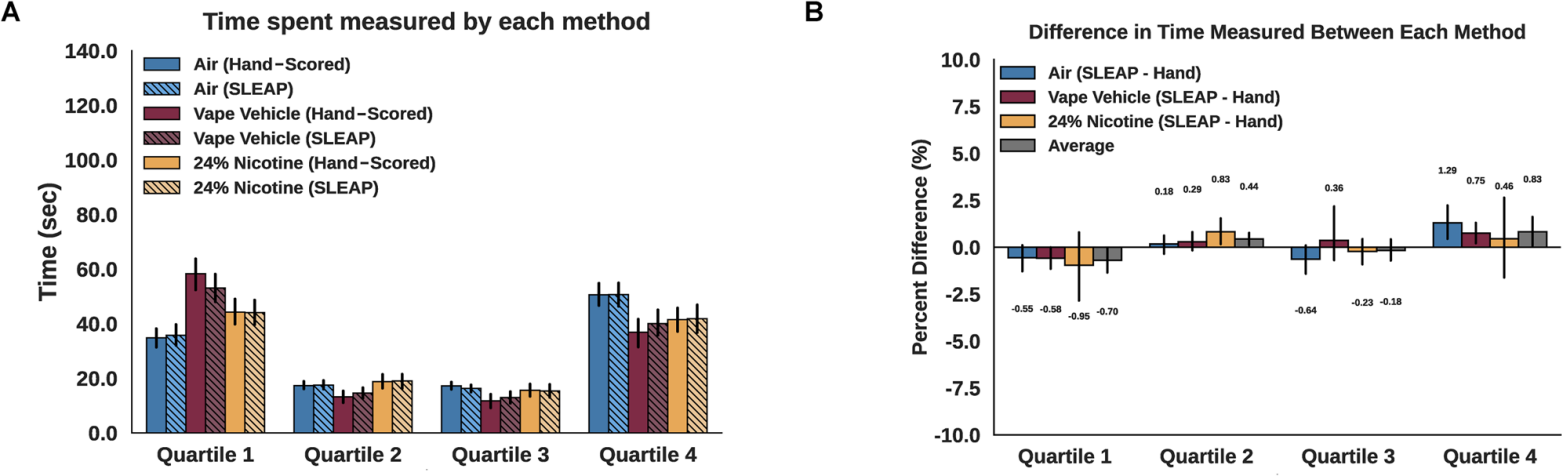
Quantitative comparison of hand-scoring and SLEAP. ***A***, No significant differences were found between hand-scored and SLEAP-scored time spent in each quadrant for each group (*t* test, *p* = 0.951). ***B***, Normalized differences between hand-scored and SLEAP-scored time spent in each quadrant. Differences are divided by 120 s to reflect 2 min recordings so that differences are reflected as percent of video length. We calculated normalized differences between hand and SLEAP scoring: 0.78% in Q3 (*t* test, *p* = 0.728), 1.54% in Q1 (*t* test, *p* = 0.688), 0.57% in Q2 (*t* test, *p* = 0.658), and 1.26% in Q4 (*t* test, *p* = 0.648). Maximum differences were observed in quartile 4 for roaches receiving air with SLEAP-scored overestimating by 1.29%. The average of all absolute errors across all quadrants was 0.59%.

We found that SLEAP was on average 0.59% different from hand-scored data of time spent in each quartile across all groups. No significant differences were found between hand-scored and SLEAP analyzed data across all quadrants (Fig. 8*A*). Focusing on specific quadrants, we found normalized differences between hand and SLEAP scoring were 0.78% in Q3, 1.54% in Q1, 0.57% in Q2, and 1.26% in Q4, but no significant differences were found between hand-scored and SLEAP analyzed data (Fig. 8*B*).

**Movie 1.** Video of roach walking with SLEAP tracks overlaid. [View online]

### Access to machine learning teaching tools

All data and resources used in the production of our machine learning model are free to access through our GitHub (https://github.com/verpeutlab/SLEAPing-with-cockroaches) or Linktree (https://linktr.ee/sleapingroaches), where a learning kit is available for educators or students to benefit. Both our GitHub and Linktree provides access to sample videos, education materials, trained models, analysis code, and guides. It is recommended that educators or students hoping to introduce themselves to machine learning in neuroscience access these links and go through our starter kit, which includes step-by-step instructions on replicating our computational methods. When the SLEAP learning kit was tested in two workshops (*n* = 38 participants), survey results concluded that participants had an increase of 94% in SLEAP knowledge and 88% would recommend using SLEAP. These workshops invited individuals from multiple institutions using email and had a combination of educators, postdoctoral researchers, undergraduate, high school, and graduate students. The feedback from these workshops was incorporated into the instruction manual of the SLEAP starter manual.

## Discussion

Here we have developed and demonstrated a low-cost educational machine learning program to use with invertebrate cockroaches to study behavior. In doing so, we created (1) a low-cost behavior testing apparatus to study cockroach place preference and addiction-like properties, (2) implementation of a machine learning program (SLEAP) to study cockroach behavior, and (3) a cloud-based software program using Google Colab to analyze data output from SLEAP that can be used in the classroom without the need for a GPU. We found that vape-exposed roaches exhibited more time spent in Q1 compared with Q4, where vape exposure was highest. The fact that roaches exposed to the vape vehicle spent more time away from the pump suggests an aversive quality of the vape vehicle. Interestingly, roaches exposed to nicotine displayed nearly opposite behavior compared with roaches receiving air. Distance metrics obtained from machine learning data and analysis also corroborate this finding. We postulate that the small differences in ingredients between the commercially available vape and nicotine drove these differences. This was also noted by others using mass spectrometry analysis of commercially available nicotine solutions. Nevertheless, the aim of this project was not only to evaluate whether roaches could be used to study addiction, but to assess the viability of machine learning methods as a means of allowing such analyses.

We successfully implemented SLEAP and found a near 0.59% difference compared with hand-scoring. Given that no significant differences in average time spent were found between scoring methods for all groups in all quadrants, we propose that this pipeline is accurate to implement in this particular experiment. An advantage of machine learning is the rich quantitative analyses possible, such as node velocity assessment, distance measurements, and behavior clustering, which provide robust information regarding individual and group behaviors. Our proposed pipeline of using SLEAP on raw video files, batch analyzing data, and performing group comparisons allowed the allocation of personnel time toward intriguing data analysis and interpretation rather than repetitive behavior scoring. Furthermore, the freely available code platform, Google Colab, is compatible with devices without a GPU and is cloud based, which encourages flexible use of analysis code across multiple devices and settings. In addition, data management is seamless across multiple users and devices using Google Workspaces that can directly access data stored in Google Sheets, assess videos stored in Google Drive, and save various SLEAP output files directly to Google Drive folders. These methods circumvent requiring files to be downloaded to local devices and allowing teams to work together simultaneously.

Most notably, velocity data of the roach itself and individual nodes provided detailed quantitative analysis to further capture individual behavior. For instance, nodes that tend to move faster, such as antennas, relative to other nodes can signify their importance in sensing environmental cues. This data was represented as a heat map (Fig. 2*D*) and can elucidate preference spots in a variety of chamber designs to guide researchers toward analyses in particular regions of interest. However, one of the most powerful utilizations of velocity metrics is the clustering of behavior (Fig. 7*A*,*B*). Using unsupervised clustering methods, such as *K*-means, allows for behavior clustering not possible by human scoring. Although supervised behavior clustering programs can elucidate various aspects of known behaviors, such as DeepEthogram (Bohnslav et al., 2021), Janelia Automatic Animal Behavior Annotator (JAABA, Kabra et al., 2013), or Simple Behavioral Analysis (SimBa; Nilsson et al., 2020), unsupervised behavior clustering allows discovery of behavior difficult to discern by the human eye. Unsupervised behavior clustering programs (i.e., ASoid; Tillmann et al., 2024) allow for classification of similar pose and velocities that comprise behavior allowing for an unbiased screen. Ethnographic data can be visualized in 3D spaces or reduced to 2D spaces using dimensionality reduction techniques (Fig. 7). Our implementation of PCA and UMAP to visualize global and local structures, respectively, demonstrates the complex data interpretation possible with machine learning. PCA and UMAP clusters visualize behavior in a manner more accessible for students to interpret: the closer the clusters, the more similar the behavior. This code was validated for function with random selections of roaches from different groups and on different days. Roaches from all groups were able to be analyzed with our code. Although we provide a wide variety of sample metrics to analyze, further analyses and visualization are only limited by the creativity and coding experience of the user.

Open-source tools and software are the future of teaching neuroscience techniques, and we found very receptive and interested audiences when testing this program. Learning how to use a machine learning program brought students out of their comfort zones, and they became more assertive in using their computers and Python as a coding language. Yet, at this time there are still several concerns to consider as others try to implement our program. First, choosing the proper camera and setup for video recording for machine learning (Robie et al., 2017) is critical, and while the simplicities of using a smartphone are numerous, smartphone cameras typically have a lower fps than a typical machine learning camera (<60 vs >200 fps) which may miss very fast behaviors (i.e., grooming). Next, downloading SLEAP and general computer use can be a barrier for some. Therefore, the ability to label nodes on an organism using a cloud-based program would be ideal. Last, there is a small amount of coding involved with using this software and while our educational program helps a novice computer programmer, having some general coding knowledge is beneficial. Therefore, we believe this simplified machine learning educational program using SLEAP will inspire students to learn more about coding and to engage sooner with research and mathematics.

In conclusion, we have designed a simple test to use in the classroom with a common neuroscience model, the cockroach, and an open-source software package to analyze behavior using machine learning. A beginner starter kit was created to allow individuals to experiment with data analysis and learn SLEAP. This kit includes original roach videos, an introduction guide, sample SLEAP tracks, sample predictions, analysis code, and sample visualizations. All materials are completely free, open-source, and require only a laptop to operate. This program could be extended to other models, such as plants, worms, and even humans. Using this program, students learn Python, data analysis, figure creation, and how to design and develop a scientific experiment. While this program meets the goals for expanding education in Arizona, it is expected that educators will be able to use these tools across the world. Lastly, these open-source tools are the future for rural schools (Høye et al., 2021), whereby resources are extremely limited, and students will be more prepared for college and increase their chances at a successful future.

## Data Availability

The code/software used to analyze the data presented here is freely available online at https://github.com/verpeutlab/SLEAPing-with-cockroaches and ASU data repository: https://doi.org/10.48349/ASU/W2E1YE.

10.1523/ENEURO.0173-24.2024.d1 Statistics Download Statistics, XLS file.

10.1523/ENEURO.0173-24.2024.d2 Code Download Code, ZIP file.

## Synthesis

Reviewing Editor: Niraj Desai, National Institute of Neurological Disorders and Stroke Decisions are customarily a result of the Reviewing Editor and the peer reviewers coming together and discussing their recommendations until a consensus is reached. When revisions are invited, a fact-based synthesis statement explaining their decision and outlining what is needed to prepare a revision will be listed below. The following reviewer(s) agreed to reveal their identity: NONE.

Thank you for revising your manuscript so well and for submitting it to eNeuro. I am sure many people -- both scientists and educators -- will benefit.

REVIEWER COMMENTS

The authors have greatly improved the manuscript, which is very readable and provides an important contribution. The inclusion of the past workshops and instructions for ease of use make this a wonderful read, and I'm sure many scientists, educators, and students at all levels will find this of interest.
