# Supplementary figures and images for: Low-Cost Approaches in Neuroscience to Teach Machine Learning Using a Cockroach Model
Source: eNeuro. 2024 Dec 12;11(12):ENEURO.0173-24.2024. doi: 10.1523/ENEURO.0173-24.2024 (PMC11654359; doi:10.1523/ENEURO.0173-24.2024)

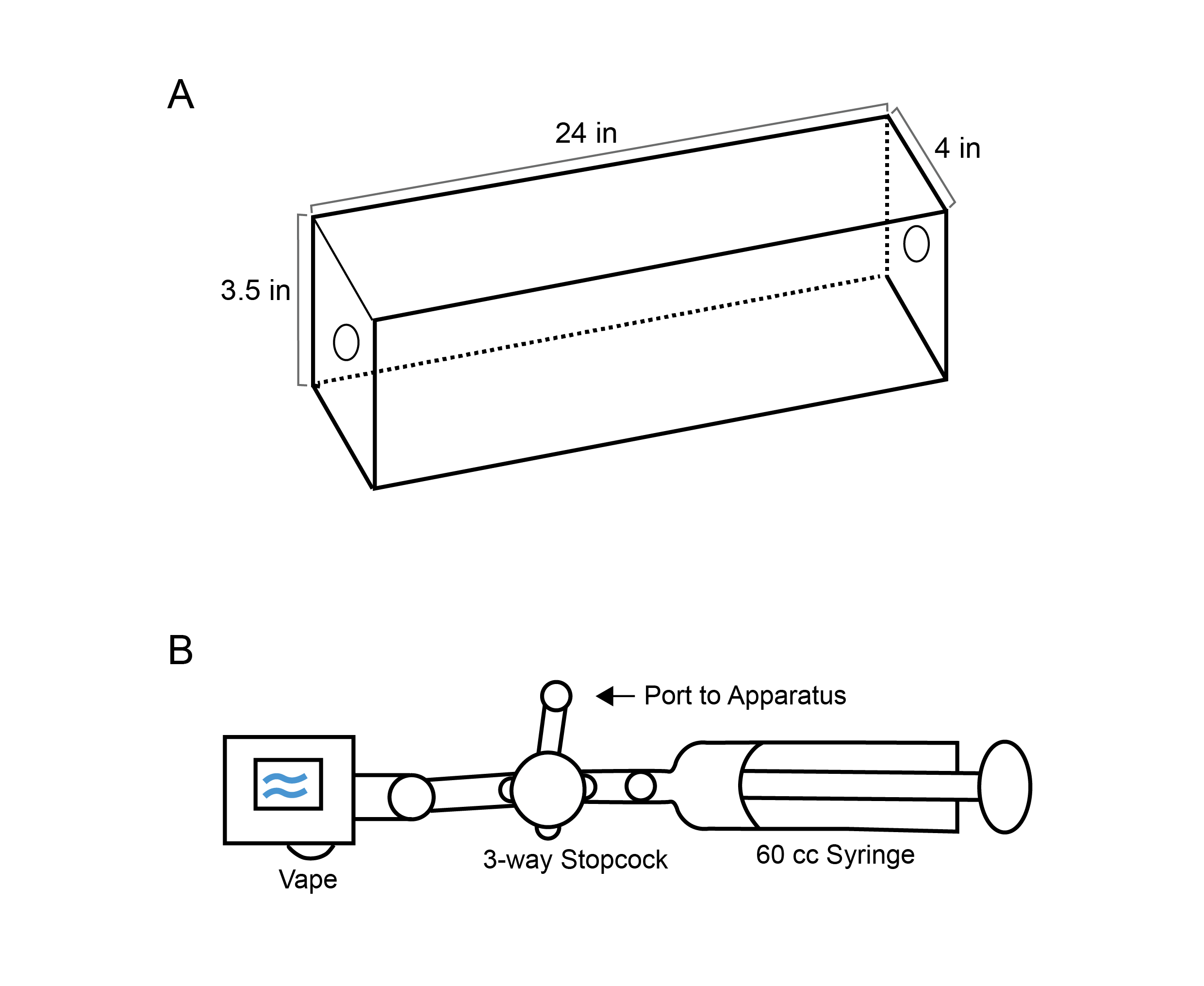

Supplement: Figure 1-1 — Customized apparatus for testing cockroach behavior after exposure to air, vape, or nicotine. A) Linear track measuring 24 in x 4 in x 2.5 in (length x width x height). B) Syringe pump connected to apparatus that delivers vape to the linear track. Download Figure 1-1, TIF file. [file eneuro-11-ENEURO.0173-24.2024-s005.tif]

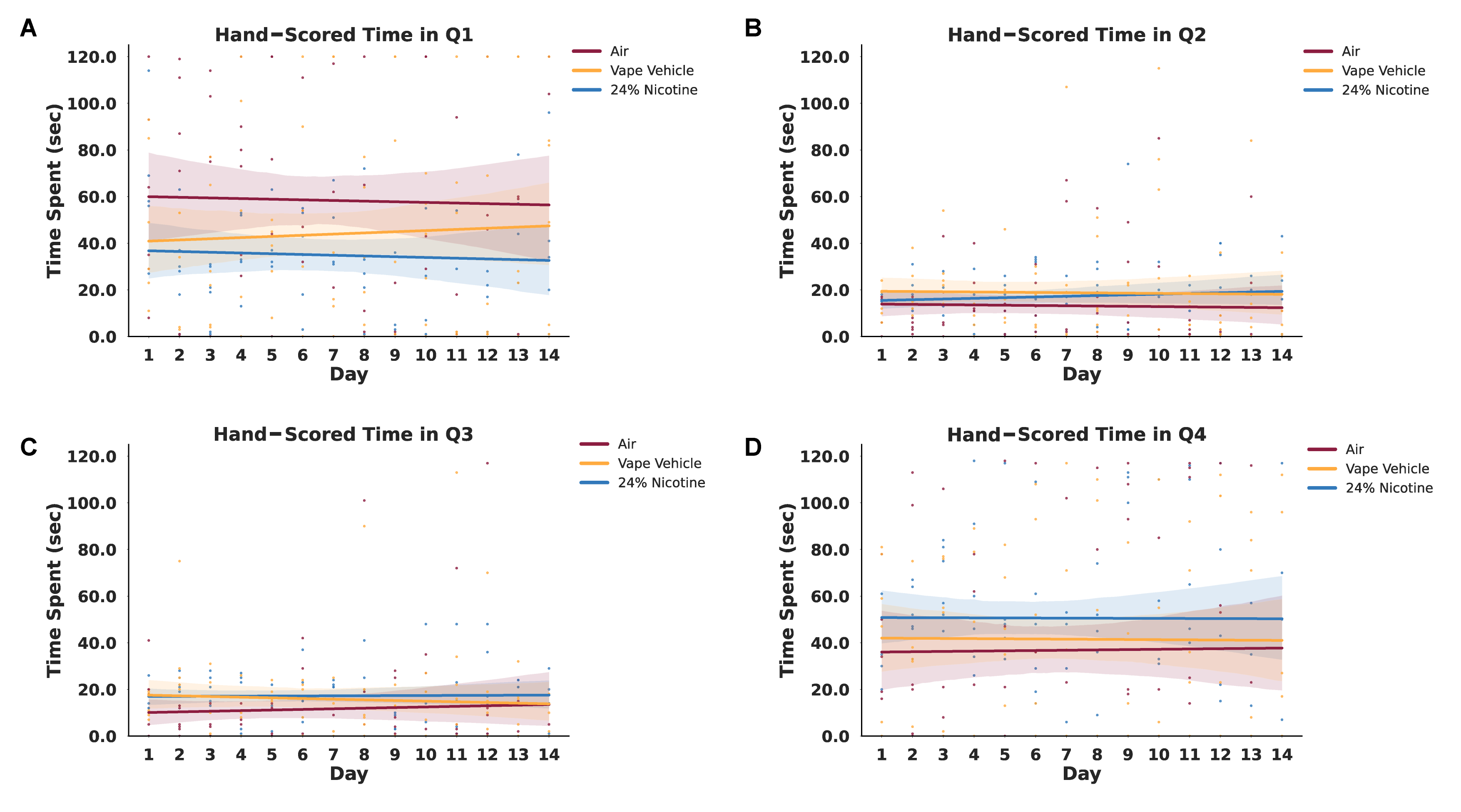

Supplement: Figure 4-1 — Simple linear regression of day versus time spent in a quadrant across 14 days using hand-scored data. We found a simple linear model does not fit our data. A) In quadrant 1, roaches exposed to air, vape vehicle, or nicotine display no differences (p = 0.98, ANOVA). Air-exposed roaches resulted in a regression line with the equation y = -0.32x + 37.09 (r2 < 0.001). Roaches exposed to vape vehicle resulted in a regression line with the equation y = -0.27x + 60.25 (r2 < 0.001). Roaches exposed to 24% nicotine resulted in a regression line with the equation y = 0.50x + 40.41 (r2 < 0.001). B) In quadrant 2, roaches exposed to air, vape vehicle, or nicotine display no differences (p = 0.93, ANOVA). Air-exposed roaches resulted in a regression line with the equation y = 0.30x + 15.19 (r2 < 0.001. Roaches exposed to vape vehicle resulted in a regression line with the equation y = -0.12x + 14.01 (r2 < 0.001). Roaches exposed to 24% nicotine resulted in a regression line with the equation y = -0.10x + 19.50 (r2 < 0.001). C) In quadrant 3, roaches exposed to air, vape vehicle, or nicotine display no differences (p = 0.99, ANOVA). Air-exposed roaches resulted in a regression line with the equation 0.04x + 16.93 (r2 < 0.001). Roaches exposed to vape vehicle resulted in a regression line with the equation y = 0.26x + 9.84 (r2 < 0.001). Roaches exposed to 24% nicotine resulted in a regression line with the equation y = -0.29x + 17.81 (r2 < 0.001). D) In quadrant 4, roaches exposed to air, vape vehicle, or nicotine display no differences (p = 0.96, ANOVA). Air-exposed roaches resulted in a regression line with the equation y = -0.04x + 50.83 (r2 < 0.001). Roaches exposed to vape vehicle resulted in a regression line with the equation y = 0.13x + 35.89 (r2 < 0.001). Roaches exposed to 24% nicotine resulted in a regression line with the equation y = 0.001x + 41.68 (r2 < 0.001). Download Figure 4-1, TIF file. [file eneuro-11-ENEURO.0173-24.2024-s006.tif]

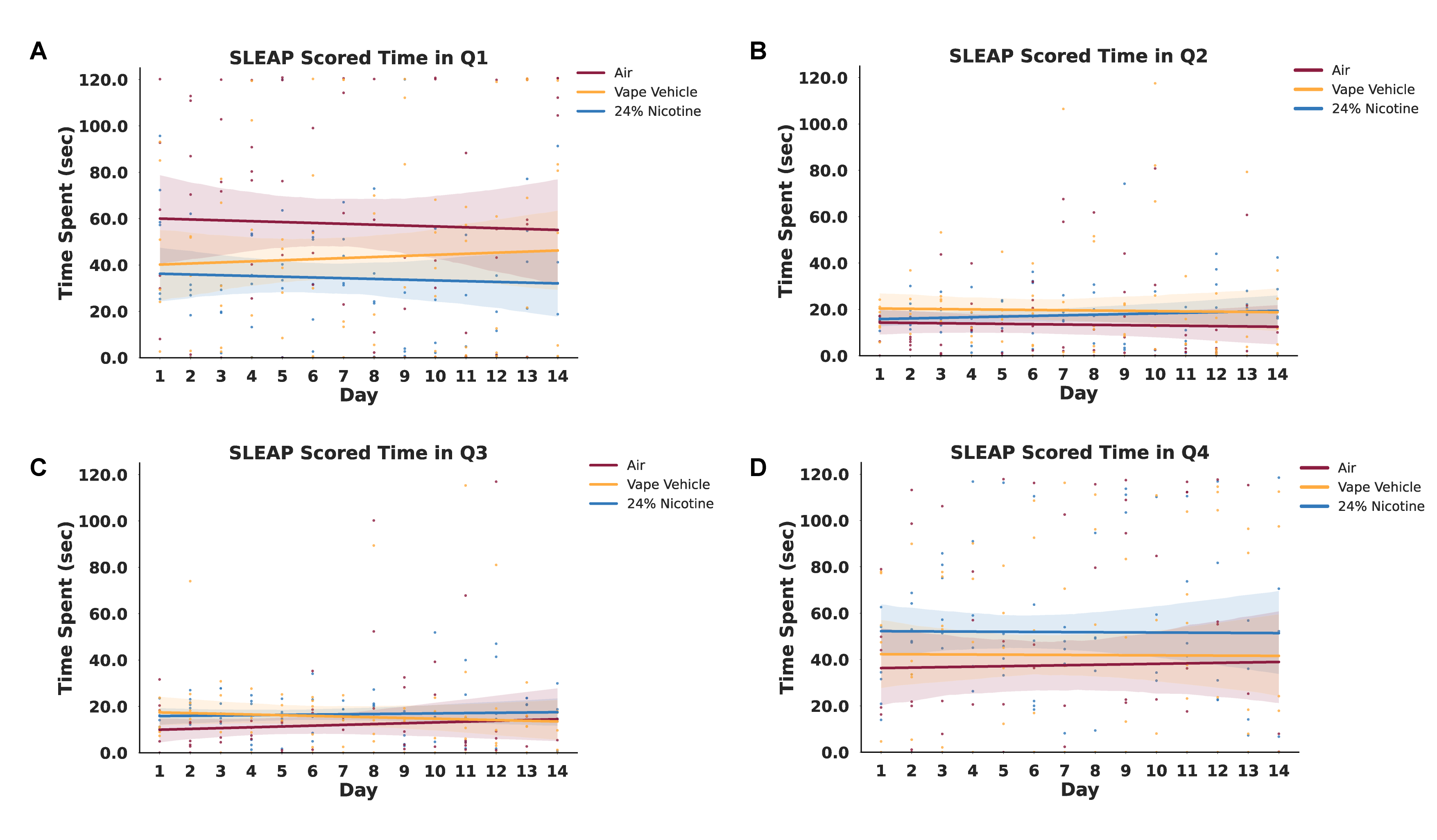

Supplement: Figure 4-2 — Simple linear regression of day versus time spent in a quadrant across 14 days using SLEAP scored data. We found a simple linear model does not fit our data. A) In quadrant 1, roaches exposed to air, vape vehicle, or nicotine display no differences (p = 0.92, ANOVA). Air-exposed roaches resulted in a regression line with the equation y = -0.32x + 36.56 (r2 < 0.001). Roaches exposed to vape vehicle resulted in a regression line with the equation y = -0.38x + 60.41 (r2 < 0.001). Roaches exposed to 24% nicotine resulted in a regression line with the equation y = 0.47x + 39.73 (r2 < 0.001). B) In quadrant 2, roaches exposed to air, vape vehicle, or nicotine display no differences (p = 0.99, ANOVA). Air-exposed roaches resulted in a regression line with the equation y = 0.27x + 15.56 (r2 < 0.001. Roaches exposed to vape vehicle resulted in a regression line with the equation y = -0.14x + 14.45 (r2 < 0.001). Roaches exposed to 24% nicotine resulted in a regression line with the equation y = -0.12x + 20.50 (r2 < 0.001). C) In quadrant 3, roaches exposed to air, vape vehicle, or nicotine display no differences (p = 0.87, ANOVA). Air-exposed roaches resulted in a regression line with the equation 0.13x + 15.70 (r2 < 0.001). Roaches exposed to vape vehicle resulted in a regression line with the equation y = 0.35x + 9.58 (r2 < 0.001). Roaches exposed to 24% nicotine resulted in a regression line with the equation y = -0.30x + 17.70 (r2 < 0.001). D) In quadrant 4, roaches exposed to air, vape vehicle, or nicotine display no differences (p = 0.99, ANOVA). Air-exposed roaches resulted in a regression line with the equation y = -0.06x + 52.31 (r2 < 0.001). Roaches exposed to vape vehicle resulted in a regression line with the equation y = 0.16x + 36.35 (r2 < 0.001). Roaches exposed to 24% nicotine resulted in a regression line with the equation y = -0.06x + 42.39 (r2 < 0.001). Download Figure 4-2, TIF file. [file eneuro-11-ENEURO.0173-24.2024-s007.tif]

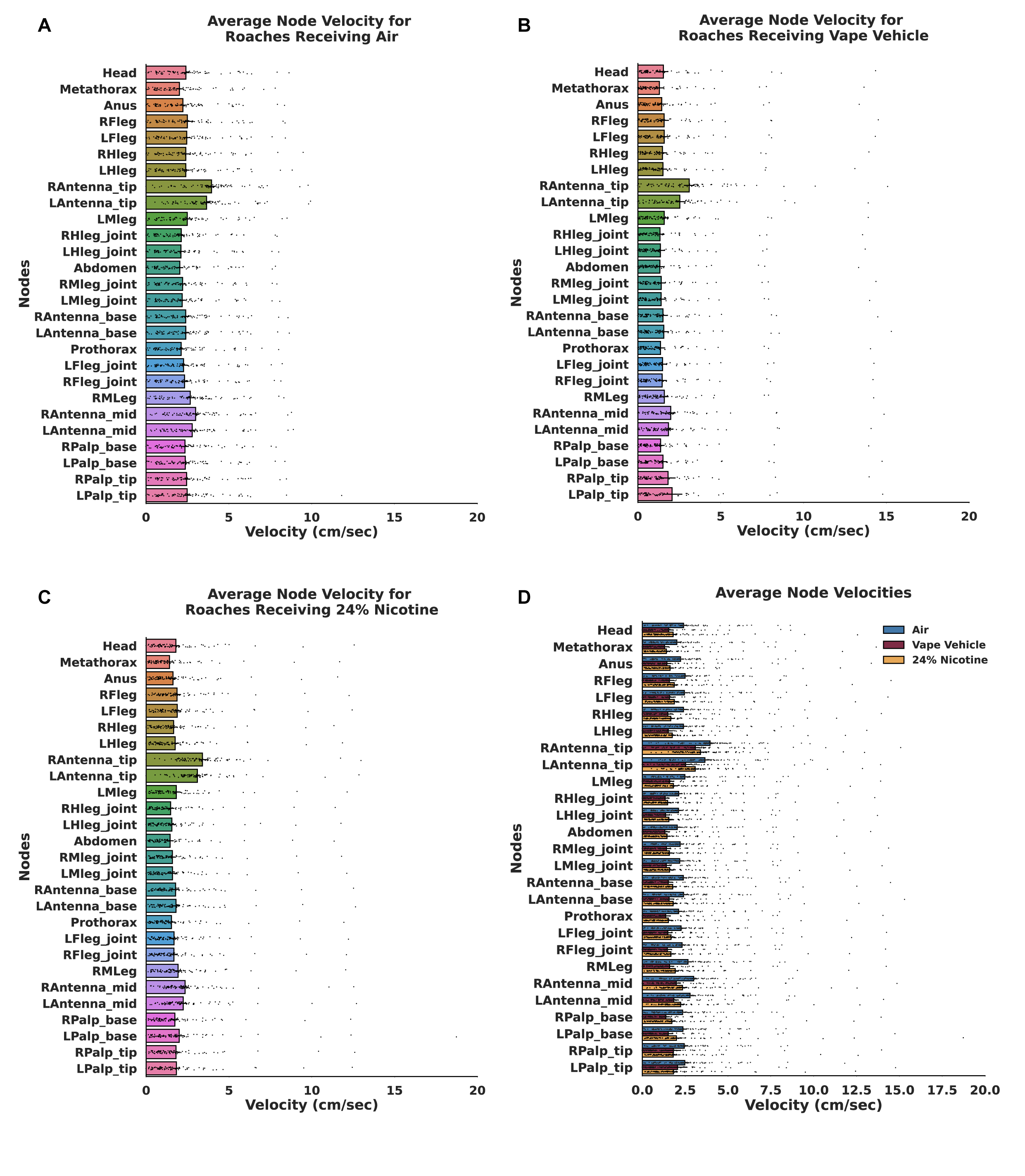

Supplement: Figure 6-1 — Average node velocity. Velocity of each node was obtained for each roach and averaged within a single group. Velocity was measured using X-Y positional data associated with certain frames obtained from SLEAP metrics. Frames were converted to seconds using the frame per second rate of the camera. A) Average node velocity for roaches exposed to air. B) Average node velocity for roaches exposed to 0% vape vehicle. C) Average node velocity for roaches exposed to 24% nicotine. D) Velocity of each node comparing between groups. Averaging all nodes across all days indicates that air-exposed roaches moved faster compared to vape (p < 0.001) or 24% nicotine (p < 0.001) conditions. Download Figure 6-1, TIF file. [file eneuro-11-ENEURO.0173-24.2024-s004.tif]
